# Whole genome copy number analyses reveal a highly aberrant genome in *TP53* mutant lung adenocarcinoma tumors

**DOI:** 10.1186/s12885-021-08811-7

**Published:** 2021-10-09

**Authors:** Maria Moksnes Bjaanæs, Gro Nilsen, Ann Rita Halvorsen, Hege G. Russnes, Steinar Solberg, Lars Jørgensen, Odd Terje Brustugun, Ole Christian Lingjærde, Åslaug Helland

**Affiliations:** 1 Department of Cancer Genetics, Institute for Cancer Research, Oslo University Hospital-The Norwegian Radium Hospital, Oslo, Norway; 2 Department of Oncology, Oslo University Hospital, 4950 Nydalen Oslo, Norway; 3 Department of Computer Science, University of Oslo, Oslo, Norway; 4 Centre for Cancer Biomedicine, Faculty of Medicine, University of Oslo, Oslo, Norway; 5 Department of Pathology, Oslo University Hospital, Oslo, Norway; 6 Department of Cardiothoracic Surgery, Oslo University Hospital, Oslo, Norway; 7 Section of Oncology, Vestre Viken Hospital, Drammen, Norway

**Keywords:** Copy number, Lung cancer, NSCLC, p53, mTOR

## Abstract

**Background:**

Genetic alterations are common in non-small cell lung cancer (NSCLC), and DNA mutations and translocations are targets for therapy. Copy number aberrations occur frequently in NSCLC tumors and may influence gene expression and further alter signaling pathways. In this study we aimed to characterize the genomic architecture of NSCLC tumors and to identify genomic differences between tumors stratified by histology and mutation status. Furthermore, we sought to integrate DNA copy number data with mRNA expression to find genes with expression putatively regulated by copy number aberrations and the oncogenic pathways associated with these affected genes.

**Methods:**

Copy number data were obtained from 190 resected early-stage NSCLC tumors and gene expression data were available from 113 of the adenocarcinomas. Clinical and histopathological data were known, and *EGFR*-, *KRAS*- and *TP53* mutation status was determined. Allele-specific copy number profiles were calculated using ASCAT, and regional copy number aberration were subsequently obtained and analyzed jointly with the gene expression data.

**Results:**

The NSCLC tumors tissue displayed overall complex DNA copy number profiles with numerous recurrent aberrations. Despite histological differences, tissue samples from squamous cell carcinomas and adenocarcinomas had remarkably similar copy number patterns. The *TP53*-mutated lung adenocarcinomas displayed a highly aberrant genome, with significantly altered copy number profiles including gains, losses and focal complex events. The *EGFR*-mutant lung adenocarcinomas had specific arm-wise aberrations particularly at chromosome7p and 9q. A large number of genes displayed correlation between copy number and expression level, and the PI(3)K-mTOR pathway was highly enriched for such genes.

**Conclusions:**

The genomic architecture in NSCLC tumors is complex, and particularly *TP53*-mutated lung adenocarcinomas displayed highly aberrant copy number profiles. We suggest to always include *TP53*-mutation status when studying copy number aberrations in NSCLC tumors. Copy number may further impact gene expression and alter cellular signaling pathways.

**Supplementary Information:**

The online version contains supplementary material available at 10.1186/s12885-021-08811-7.

## Introduction

Lung cancer is the most frequent cancer among men and the third most frequent cancer type among women worldwide [1]. Patients diagnosed with lung cancer have a high mortality, and the disease causes almost as many lost life-years as colon, prostate and breast cancer combined [2]. Non-small cell lung cancer (NSCLC) accounts for about 85% of all lung cancer cases, and tumors with adenocarcinoma histology are increasing in incidence. Genetic alterations found in lung adenocarcinomas are clinically important, and *EGFR* mutations and translocations involving *ALK, ROS* and RET genes are currently targets for therapy [3, 4]. *TP53* mutations are seen in approximately 50% of NSCLC [5], and the potential predictive and prognostic value of *TP53* mutation status is debated [6]. Chromosomal abnormalities are frequent events in NSCLC tumors, and both mutations and copy number aberrations can be main drivers of the disease [7]. Specific patterns of copy number gains and losses have been associated with different cancer types [8–10], and linked to histological subtypes of lung cancer tumors [11]. In breast cancer tumors, focal complex events characterized by multiple closely spaced aberrations seen in genome-wide copy number profiles, have been described and associated with prognosis [12]. Focal complex events has been reported to be more frequent in lung adenocarcinomas compared with other histological subtypes of lung cancer tumors [11], but thorough studies of chromosomal architecture in NSCLC tumors are lacking. Different copy number profiles in lung adenocarcinoma tumors with and without mutations in *EGFR* or *KRAS* have been described [13–15], but information about structural events have not been included in these studies. The effect of copy number aberrations on carcinogenesis is complex and some reports have shown that the expression of genes located in chromosomal regions involved in copy number alterations varies consistently with the DNA copy number [16] suggesting that these alterations can affect the expression of oncogenes and/or tumor suppressor genes [8, 9]. The affected genes may further act together to alter cellular signaling pathways in the malignant cells [17, 18].

In this study, we aimed to characterize the genomic architecture of the NSCLC tumors. Copy number data were obtained by high-resolution SNP arrays on 190 tumor samples from operable NSCLC patients. We further analyzed the complexity of the tumor genomes based on the allele-specific copy number profiles. Substantial differences were found between subgroups of samples when stratified on the basis on histology, smoking history, *EGFR-*, *KRAS-* and particularly *TP53* mutation status. Furthermore, by integrating gene expression data from a subset of 113 lung adenocarcinoma samples, we identified genes for which the expression was affected by copy number and subsequently identified the cellular pathways most enriched for such genes.

## Material and methods

### Ethic statement and patients included in the study

This project was approved by the institutional review board and regional ethics committee (S-05307). Participants included were patients with operable lung cancer admitted to the cardio-thoracic surgery department at Oslo university hospital-Rikshospitalet, from 2006 to 2011. All patients received oral and written information about the project and signed a written consent before entering the study. Clinical data were obtained from questionnaires, medical records, histology reports and the Cancer registry of Norway.

The tumor tissue was snap frozen in liquid nitrogen and stored at − 80 °C until DNA and RNA isolation. Genomic DNA was extracted from the frozen tumor tissue using the Maxwell® 16 DNA purification kit following standard protocol and RNA was extracted with standard TRIZOL methods (Invitrogen, Carlsbad, CA, USA) as previously described [19, 20]. *EGFR* mutation analyses of exons 18–21 were performed by using the TheraScreen EGFR mutation kit and the *KRAS* mutations were tested by using the wobble-enhanced ARMS (WE-ARMS) method [21]. *TP53* mutations in exon 2–11 were analyzed by Sanger sequencing using the AB 3730 DNA Analyzer (Applied Biosystems) after standard protocol as previously described [20].

### SNP arrays and mRNA expression arrays

DNA was hybridized to Affymetrix Genome-Wide Human SNP 6.0 arrays following the manufacturer’s instructions (Affymetrix, Santa Clara, CA) at AROS Applied Biotechnology (Aarhus, Denmark). A subset of the lung adenocarcinoma samples (*n* = 113) had mRNA expression data available. This was assessed using gene expression microarrays from Agilent technologies (SurePrint G3 human GE, 8 × 60 K) as previously described [20]. The mRNA data were log2-transformed and normalized between arrays by using the 75th percentile method in Genespring GX analysis Software v.12.1 (Agilent technology). The mRNA expression array includes 42,066 unique probes, and 30,370 probes remained after filtering out probes with no gene annotation or available gene names. The average gene expression value was calculated when a gene was mapped with more than one probe at the array. 22,076 unique genes remained for further analyses.

### Statistical analyses

#### Copy number segmentation and estimation of allele-specific copy numbers

The SNP data (Affymetrix CEL-files) were pre-processed by using the Affymetrix Power Tools (APT) software and the PennCNV software [22] to obtain total signal intensities (LogR) and B allele frequencies (BAF) at each genomic marker. All samples were normalized to a custom-made cohort of normal samples from the HapMap project, the 1000 Genomes Project and the Wellcome Trust Case-Control Consortium [23–25]. After adjusting LogR for GC binding artifacts [26], the LogR and BAF results were used as input for the allele-specific segmentation of normalized raw data with ASPCF [27] with penalty parameter γ = 50 and the subsequent analysis with the ASCAT (Allele-Specific Copy number Analysis of Tumors) algorithm (version 2.3) [28]. The result was an allele-specific copy number profile of each tumor as well as estimates of tumor ploidy and tumor cell fraction (cellularity). In five samples, ASCAT failed to come up with a solution, and these samples were excluded from further analyses.

#### Identification of recurrently aberrant regions

From ASCAT we obtain for each sample a segmentation of the tumor genome, where each segment has a start and end position in the genome and two allele-specific copy number values (0, 1, 2, .....) corresponding to the two alleles. The sum of the two copy number values represents the total copy number of the segment, and the median total copy number across the whole genome (taking into account the size of each genomic segment) represents a measure of tumor ploidy. Segments with total copy number exceeding the ploidy were called as gains, while segments with total copy number less than the ploidy were called as losses. The identified aberrations among all NSCLC samples were plotted with the frequency of samples with segments called as gain or loss (in the y-axis) at every genomic position (x-axis). Segments that were aberrant in a fixed proportion p of the samples were defined as recurrent aberrant regions and were matched by position to an annotation file with information of gene names, cytobands and chromosomal regions (in this paper, we used *p* = 0.30).

#### Quantification of genomic complexity from copy numbers

**Fig. 1:**
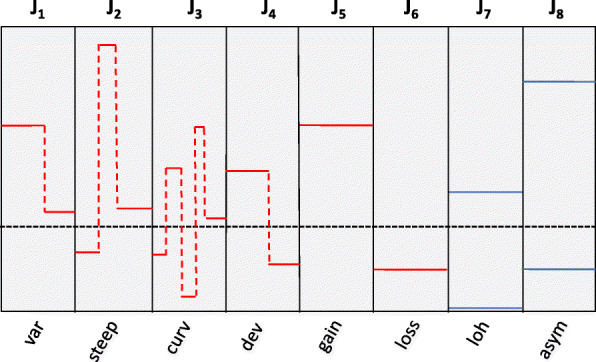
An overview of the eight indices in the algorithm. Var: variation relative to median copy number (J1), steep: steep transitions (J2), curv: curvature or oscillation (J3), dev: deviation from ploidy (J4), gain: gain relative to ploidy (J5), loss: loss relative to ploidy (J6), loh: LOH related allelic skewness (J7), and asym: non-LOH related allelic skewness (J8). The scores can be detected genome-wide or arm-wise in each sample. The red curves represent total copy numbers, while the blue curves represent the number of copies of each allele. Ploidy is defined as the median genome copy number and is shown as a dashed horizontal line

To more thoroughly examine the segmental and structural DNA aberrations, we applied a bioinformatic method that compresses the allele-specific copy number profile into a small number of score values capturing the degree of presence of features in the profile. The algorithm compresses the allele-specific copy number profile in a specified genomic region R into eight scores that reflect different aspects of the genomic complexity. In this paper, the genomic region R was either the whole genome (resulting in eight scores per tumor) or a chromosome arm (resulting in 8 × 43 = 344 scores per tumor when applied to all 43 arms separately). The scores reflect level of variation relative to the median copy number in the region (*var*), level of steep transition in copy number (*steep*), level of curvature or oscillation in copy number (*curv*), level of deviation from the genome ploidy (*dev*), level of gain relative to ploidy (*gain*), level of loss relative to ploidy (*loss*), level of allelic skewness attributable to loss of heterozygosity (*loh*), and level of non-LOH related allelic skewness (*asym*). Figure 1 gives a visual guide to the type of features this algorithm scores can capture in a copy number profile. Note that the first six scores are calculated based on total copy numbers, while the last two use the allele-specific copy numbers. All scores take the magnitude as well as the width of aberrant regions into account. The first score, *var,* will be large if there are local variations in copy number. The score *steep* reflect if there are narrow copy number shifts of some magnitude, while the score *curv* reflects such events that are also oscillating. Both *steep* and *curv* are associated with focal complex events. The fourth score, *dev*, detects the amount of deviation from the genome ploidy, while the scores *gain* and *loss* reflect whether such deviations correspond to copy number gain or loss, respectively. Note that the scores *gain* and *loss* are different from the gains and losses defined in the previous paragraph as both the magnitude and the width of the aberrant regions go into the calculation of the former whereas the latter simply reflect an indicator of whether a segment is aberrant or not. Both scores *loh* and *asym* are triggered by regions where there is skewness in the number of copies of the two alleles. The former is, however, restricted to only reflect events where one of the alleles is completely lost, while the latter detects events where this is not the case. The genome-wide and arm-wise scores were further applied to analyze the lung cancer samples stratified into defined subgroups. The bioinformatic method used in this paper is a variant of the CARMA algorithm [29].

#### Statistical hypothesis tests

Most statistical analyses were performed by using the R computing framework [30] and SPSS (v.21). T-tests were applied to perform two-sample comparisons of means, and the non-parametric Wilcoxon rank-sum test was used when the T-test was not appropriate because of strong deviations from normality of the data. *P*-values < 0.05 were considered statistically significant and Bonferroni corrections for multiple testing were performed when appropriate.

#### Integration of gene expression and copy number data; cis-associated genes

To focus on genes for which copy number is likely to affect gene expression, we integrated copy number data with mRNA expression data obtained in a subset of 113 lung adenocarcinoma samples. To obtain matched copy number and gene expression values for each sample, we found for each gene expression probe its location in the genome and the overlapping copy number segment as found by ASCAT. The value of the expression probe and the estimated total copy number of the segment then formed a matched pair, and this calculation was performed for all gene expression probes.

To identify genes likely to be substantially influenced by copy number, a combination of two criteria were used and the resulting genes were referred to as cis-genes. First, the Pearson correlation between the copy number value and the gene expression should exceed 0.4. Second, at least one of the following two t-tests should be significant (*P* < 0.05, with no adjustment for multiple comparisons). The first test compares the expression level of samples with loss and samples with normal copy number, while the second test compares the expression level of samples with normal copy number and samples with gain (where loss and gain are defined as described previously). For the purpose of visualization, the smaller of the two *P*-values was rescaled to Z-scores by applying the transformation Z = −F^− 1^(Pval), where F is the cumulative standard normal distribution. P-values are uniformly distributed between 0 and 1 and the Z-score will follow a standard normal distribution; furthermore, *P* < 0.05 corresponds to Z > -F^− 1^(0.05) = 1.64. Note that the tests above were designed to be liberal in order to also capture genes that were moderately associated with copy number; hence there was no correction for multiple comparisons above.

#### Pathway analysis

Ingenuity Systems Pathway Analysis (IPA) was applied to the list of cis-genes to derive the most enriched pathways associated with the genes regulated by copy number. A core-analysis was assessed to find the level of representation of our selected genes in already defined canonical pathways. The significance of the association between the cis-gene list and the pathways were tested with Fisher’s exact tests, and the Benjamini-Hochberg correction for multiple testing was applied.

## Results

High-resolution SNP arrays were used to obtain copy number data for 200 NSCLC samples. Five samples were excluded after pathological re-examination and five samples failed to come up with a solution in the ASCAT algorithm, leaving 190 NSCLC samples for further analyses.

The majority of samples were adenocarcinomas (*n* = 154) while squamous cell (*n* = 32) and large cell (*n* = 4) histology were also represented. To avoid picking up histological differences, only the adenocarcinoma samples were included in the subgroup and integration analyses. All the 20 *EGFR*-mutated tumors were of adenocarcinoma histology and the patients with *EGFR*-mutated tumors were mainly women (16/20) and never-smokers (12/20). The patients with *KRAS*-mutated tumors were both men (21/55) and women (34/55), and most of them were current- or former smokers (52/55). *TP53* mutations were also present in both men and women (43/79, 36/79), and 11.4% were never-smokers. The *EGFR* and *KRAS* mutations were mutually exclusive. 12 of the patients had double mutations including *KRAS* and *TP53* and 10 patients had both *EGFR*- and *TP53*-mutations. The main clinical and molecular characteristics are shown in supplementary Table 1.

### Recurrent aberrations, tumor ploidy and aberrant cell fraction

The ASCAT algorithm estimates the fraction of aberrant cells and allows the determination of tumor ploidy for each sample. The median tumor ploidy across all samples was 2.71 (range 1.51–5.49), with the highest proportion of samples with a ploidy close to 2 N, a second prominent peak at 3.5 and a third peak at 5.5 (supplementary Fig. 1). The mean estimated aberrant cell content was 53.3% (21–100%). There were no statistically significant differences in tumor ploidy or aberrant cell fraction between histological subtypes, *EGFR-*mutated/wild type, *KRAS*-mutated/wild type or stratified by stage or smoking status. The tumors with *TP53* mutations had a higher tumor ploidy (*p* = 0.012) and a smaller fraction of aberrant cells (*p* = 0.007) compared with the *TP53* wild type tumors.

**Fig. 2:**
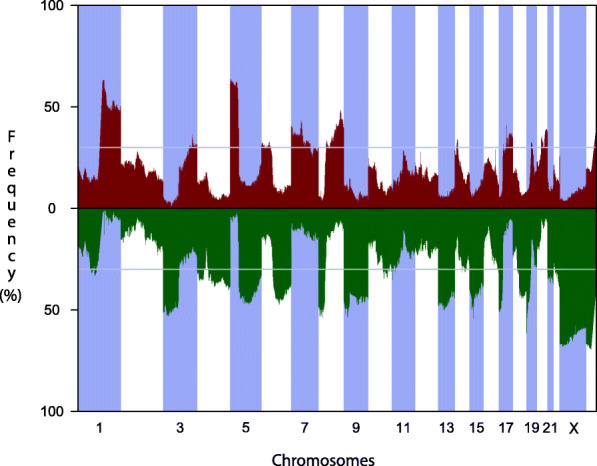
A frequency plot of all NSCLC tumors which shows the frequency of samples (in the y-axis) called to have gain (red) or loss (green) across the genome (x-axis). The dashed horizontal line indicates 30% of the samples

The NSCLC tumor tissue displayed overall complex DNA copy number profiles with numerous recurrent alterations observed in almost all chromosomes (Fig. 2). Gains were enriched in chromosome arm 1q, 3q, 5p, 6p, 7p/q, 8q, 14q, 17q, 19q and 20p/q, with chromosome arm 1q and 5p as the most frequently gained regions. Losses were located at 1p, 3p, 4p/q, 5q, 6q, 8p, 9p/q, 10q, 11p, 13q, 14q, 15q, 16q, 17p, 18q, 19p, 21q, 22q, X and Y, with 3p and X as the regions with the highest frequencies of loss. Several of the alterations were found across histological subtypes. The recurrently aberrant regions included genes implicated in the pathogenesis of NSCLC with known oncogenes such as *MDM4* (1q), *RIT1* (1q), *DROSHA* (5p), *PIK3CA* (3q), *EGFR* (7p), and *NKX2–1* (14q), and tumor suppressor genes such as *TP53* (17p). A more detailed overview of recurrent chromosomal aberrations (> 30% of the tumors) including positions, cytobands and genes located in these regions are found in supplementary Table 2, and an overview of the aberrations is listed up in supplementary Table 3.

### DNA copy number profiles between subgroups of samples

To study the differences in DNA allele-specific copy number profiles between NSCLC tumors based on histology and lung adenocarcinoma samples stratified by smoking history, *EGFR-*, *KRAS-* and *TP53* mutation status, we used genome-wide and arm-wise scores. As explained in the methods section, this algorithm compresses the allele-specific copy number profile into eight scores which captures complementary facets of the copy number profiles. Examples of arm-wise scores calculated for two selected copy number profiles are depictured in supplementary Fig. 2.

By using the genome-wide scores for all eight indices, no differences were identified between the adenocarcinomas and squamous cell carcinomas (supplementary Fig. 3a). At the arm-wise level, the histological subtypes differed at chromosome arm 1q, 3q, 5q, 6q, 12p, and 19q. The adenocarcinomas had a significantly higher gain-score at chromosome arm 1q and 5q, and a greater loss- and LOH score at 6q and 12p. The chromosome arm 3q had significantly higher gain- and asymmetry-scores in the squamous cell carcinomas. Focal complex events were observed in the adenocarcinomas at 19q (*curv*) and were combined with LOH. The *dev* score detects the amount of deviation from the genome ploidy and did also pick up the difference in copy number at chromosome arm1q and 3q (supplementary Fig. 3b and 3c).

**Fig. 3:**
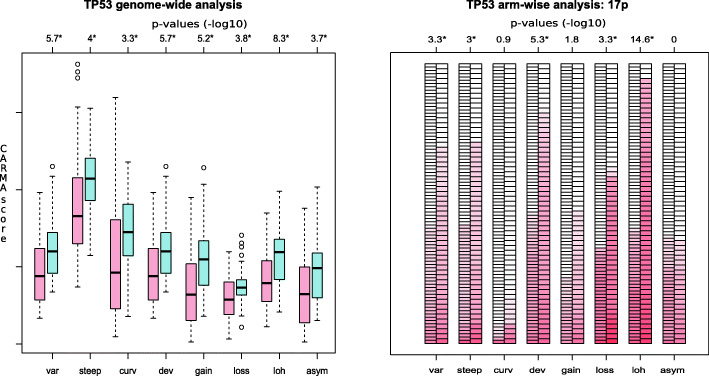
**a** All eight scores were significantly higher in the TP53 mutated tumors compared with the TP53 wild type tumors in the genome-wide analysis. The box plot shows the genome-wide scores for the TP53-mutated tumors (blue) and the TP53 wild type tumors (pink) for all eight indices. The *p*-values (−log10) from the comparison analyses are shown above each column and a value > 2 was considered as significant after multiple testing corrections. **b** The arm-wise scores at chromosome arm 17p showing the eight indices for the TP53 wild type tumors at the left and the TP53 mutated tumors at the right. The scores for each sample are colored by intensity. The p-values (−log10) from the comparison analyses are shown above each column and a value > 3 was considered as significant after multiple testing corrections

The most striking findings in the subgroup-analyses were the large difference at all genome-wide indices between the *TP53*-mutated compared with the *TP53* wild type lung adenocarcinomas (Fig. 3a). All eight genome-wide scores were significantly higher in the *TP53*-mutated lung adenocarcinomas. In the arm-wise analysis, we identified significantly higher scores in at least one index in nearly all chromosome arms (detailed information in supplementary Table 4). The chromosome arm 17p, which includes the location of *TP53*, had significantly more loss and LOH in the *TP53-*mutated lung adenocarcinomas (Fig. 3b). When we studied the exact location of the *TP53* gene at 17p, we found that 55 of the 58 lung adenocarcinomas with *TP53* mutation had loss of the wild type *TP53* locus (94.8%). The remaining mutated allele was frequently amplified in the *TP53*-mutant tumors. Of the *TP53* wild type tumors, 27 of 96 (28.1%) had *TP53* LOH, which is a significantly lower frequency than the *TP53*-mutated samples. No samples had homozygote deletion of the *TP53* gene.

**Fig. 4:**
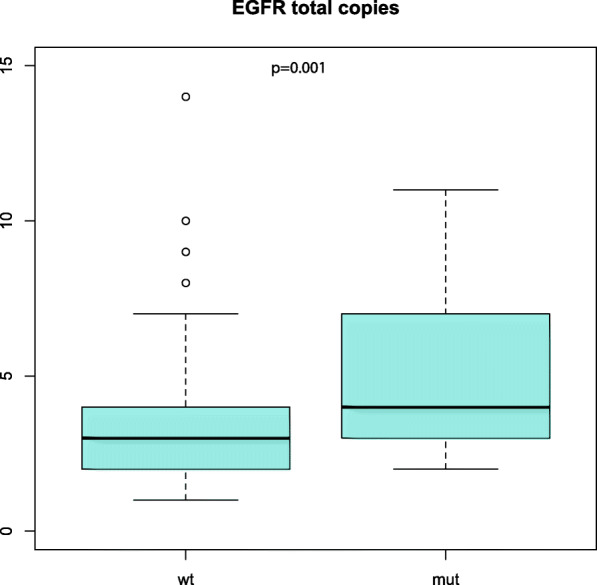
The box plot shows the total copy number at the location of the EGFR gene. The EGFR-mutant samples had significantly more copies than the EGFR wild type tumors (*p* = 0.001)

When we stratified the samples based on differences in *EGFR* mutation status, we did not identify differences at the genome-wide level (supplementary Fig. 4a). By investigating arm-wise differences, we found that the *EGFR* wild type tumors had a significantly higher loss-score at chromosome arm 7p. The gain-score at 7p was borderline significantly higher in the *EGFR* mutated tumors but was also frequently amplified in the *EGFR* wild type tumors (supplementary Fig. 4b). The *EGFR* wild type tumors had more gain at 9q and LOH in 4p and 11q, and significantly more focal complex events at 3p, 5p, 11q and 12q captured by the curv-score. Zooming in at the genomic location of the *EGFR* gene, we found that both the *EGFR*-mutated samples and the *EGFR* wild type adenocarcinomas had an increased number of copy numbers at this position, but that the *EGFR*-mutant tumors had a significantly higher number of copies compared with the *EGFR* wild type tumors (*p* = 0.001) (Fig. 4).

Stratifying the samples by *KRAS* mutation showed significantly higher genome-wide scores on *steep* and *curv* in the *KRAS* wild type tumors, reflecting generally more focal complex events in these tumors (supplementary Fig. 5). This was also seen in the arm-wise analysis with more focal complex events in the *KRAS* wild type tumors at chromosome arm 2p, 11p, 11q, and 18q. Comparing tumors from never-smoking patients and former smokers/smokers did not identify significant differences at the genome-wide level. At the arm-wise level tumors from former smokers/smokers had significantly more focal complex events at chromosome 2q and 3q. Tumors from former smoker/smokers had additionally higher loss-scores at 5q and 7p and higher gain-score at 12p.

The differences between subgroups of samples are summed up in supplementary Table 4.

### Cis-associated genes and affected pathways

**Fig. 5:**
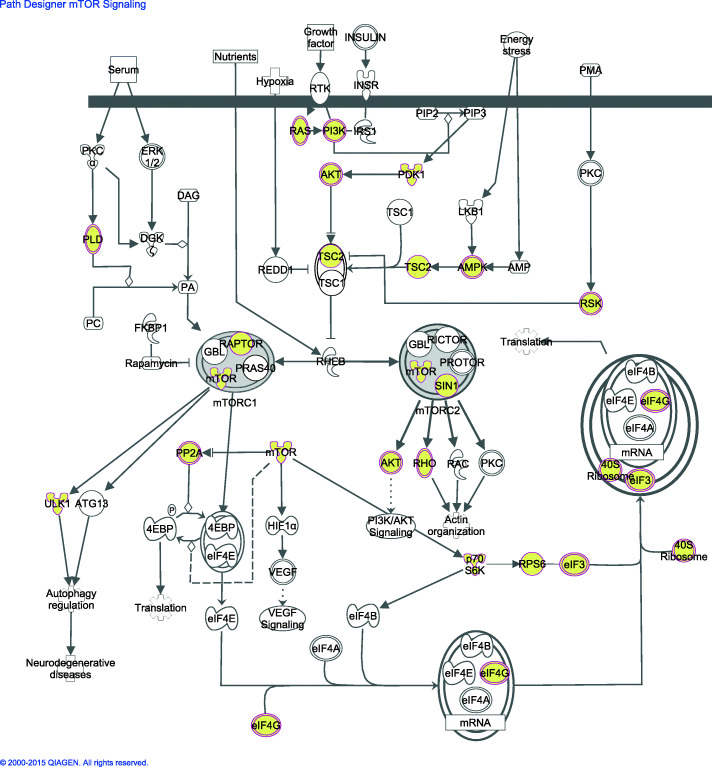
Ingenuity Pathway Analysis was used to identify cellular signaling pathways related to the identified cis-genes (genes with expression influenced by the copy number) in lung adenocarcinoma tumors. The mTOR pathway was one of the top signaling pathways in this analysis and the molecules colored yellow were among the genes identified as cis-genes

The integrative analysis was assessed to identify genes with expression influenced by the copy number. Among the 22,076 genes profiled, a total of 2868 genes abided the two criteria and were identified as cis-genes (both correlated in cis with a coefficient > 0.4 and a significant difference in gene expression between samples with copy number gain versus normal or loss versus normal) (supplementary Fig. 6). These genes were localized throughout the genome, but particularly in the recurrently aberrant regions which includes 1668 of the cis-genes identified. Genes such as *EGFR*, *PIK3CA*, *DROSHA*, *MDM4*, and *APC* were located inside recurrently aberrant regions, while other known cancer-genes such as *NF1*, *MET* and *mTOR* were identified as cis-genes and located outside the most recurrently altered regions. Using Ingenuity Pathway Analysis, signaling pathways associated with the copy number driven cis-genes were identified (supplementary Table 5). Interestingly, a large part of the cis-genes was associated with gene expression, post-transcriptional modifications and post-translational modifications, identified as the top molecular and cellular functions from the IPA-analysis. Among the top significantly associated pathways were the EIF2- (*p*-value < 1.02·10^− 8^) and the mTOR-signaling pathway (p-value < 8.82·10^− 4^), which included 57 and 45 cis-genes respectively. *PIK3CA* is a key molecule in both pathways, and this gene was identified as a cis-gene with significantly higher expression in tumors with gain compared with normal copy number at the position of the *PIK3CA* gene. In the mTOR-pathway a large number of important genes were among the identified cis-genes such as *mTOR, AKT, KRAS, RPS6, RAPTOR, EIF4G1* and *RPS6KB1 (p70S6K)* (Fig. 5). In the EIF2-pathway most of the EIF-genes (Eukaryotic translation initiation factor) were differentially expressed between the gain/normal and the ribosomal protein genes were differentially expressed between the loss/normal. 68% of the cis-genes in the EIF2 pathway and 64% of the cis-genes in the mTOR pathways were located in recurrently aberrant regions.

## Discussion

The current study presents an exploratory whole-genome investigation of copy number alterations including the genomic architecture of NSCLC tumors. The copy number data were obtained using high-resolution SNP arrays of 190 early-stage NSCLC tumors. By stratifying the samples into biological relevant subgroups, we identified large differences particularly in the TP53-mutated tumors that displayed a considerable number of gains, losses and focal complex events both in the genome-wide and arm-wise analyses. Integration of DNA copy number with mRNA expression data showed that genes with expression influenced by the copy number were associated with important cellular signaling pathways previously not known to be driven by copy number change.

Overall, the NSCLC tumor tissue displayed complex DNA copy number profiles with many gains, losses and focal complex events throughout the genome. The global copy number profile was comparable to those seen in similar studies of NSCLC tumors [10, 31, 32]. The most common regions with gains were located at chromosome arm 1q and 5p. Chromosome arm 1q includes *MDM4* and *RIT1* genes, and by integrating mRNA data, we found that expression of *MDM4* correlated with increased copy number leading to up-regulation of the expression of this gene. *MDM4* is important in carcinogenesis, and increased *MDM4* activity can suppress the *TP53* activity allowing the cancer cells to proliferate [33]. Mutation in *RIT1* has recently been identified in lung adenocarcinomas and *RIT1* is proposed to be a driver oncogene in a specific subset of lung adenocarcinomas [34]. This gene was recurrently gained in our data and may be an alternative path for oncogenic activation. The commonly gained 5p region includes *DROSHA* whose mRNA expression is correlated in-cis in our data. This gene is a crucial regulator of microRNA expression, and increased expression of *DROSHA* has been linked to poor prognosis in lung cancer [35, 36]. Other recurrently gained regions were 7p that includes the *EGFR* gene, and this region was frequently gained both in *EGFR*-mutated and wild type tumors. Chromosome arm 3p was lost in a high proportion of samples. This is a known event in lung cancer tumors, and was among the first chromosomal abnormality to be recognized originally identified by karyotyping [37].

Despite distinct histological differences, squamous cell carcinomas and adenocarcinomas show remarkable similar copy number patterns with no significant differences in genome-wide scores. In the arm-wise analysis, however, squamous cell carcinomas had a higher gain score at 3q as compared with adenocarcinomas. The squamous cell carcinomas also have significantly increased asymmetry-score at 3q, indicating an asymmetric gain in this chromosome arm. The lung adenocarcinomas had significantly more gain at chromosome arm 1q and 5q and a greater loss combined with LOH at 6q and 12p. The copy number differences between squamous cell carcinomas and the adenocarcinomas of the lung have been studied by others, and the increased gain at 3q in the squamous cell carcinomas have been reported in several studies [38, 39]. The increased gain at 5q in the lung adenocarcinomas has also been reported previously by Staaf et al. [11]. This study is the only other study which has included focal complex events in NSCLC tumors, and they identified more focal complex events in squamous cell carcinomas. This was not confirmed in our data, but the limited number of squamous cell carcinomas included in this study might serve as an explanation. Furthermore, we find that gain often occur together with asymmetry, indicating that gain often is an asymmetric event with respect to the two alleles in NSCLC tumors. Similarly, loss and LOH often co-occur when the loss is not a copy neutral event.

Mutations in the *TP53* gene are common events in lung cancer tumors*.* The *TP53*-mutated tumors had significantly higher ploidy, estimated by the ASCAT algorithm, compared with the *TP53* wild type tumors. After adjusting for estimated ploidy and aberrant cell count, we performed comparison analyses and found that the *TP53*-mutated lung adenocarcinomas had a significantly higher score at all eight indices in the genome-wide analysis (Fig. 3). The lung adenocarcinomas with mutant *TP53* gene have generally more segments deviating from the median ploidy with consistently more gains and losses throughout the genome. The *TP53*-mutant tumors additionally have more focal complex events captured by the *steep* and *curv* scores, which may be contributing to the aggressive phenotype associated with the *TP53* mutations seen in other cancer types [40]. The genome-wide LOH and asymmetry scores were additionally significantly higher in the *TP53*-mutated tumors. Particularly interesting is it that nearly all *TP53*-mutated tumors had *TP53* LOH, indicating that inactivation of both *TP53* alleles are important as proposed in Knudsons two-hit hypothesis [41]. The LOH events in *TP53*-mutated tumors were often accompanied by copy number gain of the mutant allele. In the *TP53* wild type tumors, the *TP53* LOH was seen in 31.4% of the samples, suggesting a dysfunction in the *TP53*-pathway in a large amount of all lung adenocarcinoma tumors.

The high genome-wide scores in the *TP53* mutated tumors indicate a highly unstable genome. Other studies have demonstrated how *TP53* mutation status might reflect tumor mutation burden, and association with longer overall survival in patients receiving immunotherapy [42]. This reflects that the well-known TP53 mutation status might be clinically important also in the future. The finding of the complex copy number profiles in *TP53*-mutant lung adenocarcinomas in our study is very convincing, and we suggest that *TP53*-mutation status should be considered implemented for biological stratification purposes, in studies involving genomic aberrations.

Lung adenocarcinomas with *EGFR* mutation comprise a specific clinical subtype and are more frequent in women, never-smokers and patients with Asian ethnicity [43]. To better understand the biology of the *EGFR*-mutated lung adenocarcinomas we compared copy number profiles between *EGFR*-mutated and wild type tumors. In the chromosome arm-wise analysis, we identified alterations of chromosome arm 7p that were gained in both *EGFR*-mutated and *EGFR* wild type tumors, and significantly more lost in the *EGFR* wild type tumors. The *EGFR* gene is located at 7p12, and this region was also significantly gained in both *EGFR* wild type and *EGFR*-mutated tumors, but with a significant higher number of total copies in *EGFR*-mutated tumors (Fig. 4). When integrating mRNA expression data, we found that the *EGFR* mRNA expression was correlated with gained copy number, and previous studies have also shown that the copy number alterations in chromosome 7 are correlated with protein expression and activation of the *EGFR* pathway [44]. *EGFR* mutation is a strong predictive biomarker for tyrosine kinase inhibitor response. It is however debated if copy number gain may act as a predictive marker for EGFR*-*TKI response in patients with *EGFR* wild type lung cancer tumors [45, 46]. The aberrations of chromosome arm 7p seem to be consistent with previous reports [13, 14, 47]. We also found that the *EGFR* wild type lung adenocarcinomas had significantly more gain at 9q, an aberration difference not described earlier. Other copy number differences in gains and losses between *EGFR*-mutated and wild type tumors have been described previously [13, 14, 47], but were not validated in our study. The lack of consistency may be caused by small sample sizes and the use of different methods to call gains/losses. The scores in our analyses include both the magnitude and the width of the aberrant region into the calculation of *gains* and *losses,* which makes it challenging when comparing the results with other studies. To our knowledge, focal complex events have not been described in relation to *EGFR*, *KRAS* and *TP53* mutation status, and the clinical impact of such events in lung cancer tumors is not known. The arm-wise analysis identified more focal complex events (reflected in *steep* and *curv* scores) in the *EGFR* wild type tumors with significantly higher *curv* scores at 3p, 5p, 11q and 12q. The same trend was seen in the *KRAS* wild type tumors, which both had significantly higher *steep* and *curv* scores at the genome-wide analysis and at specific chromosome arms. The *EGFR-* and *KRAS* wild type adenocarcinomas had additionally significantly more arm-wise aberrations compared with the *EGFR*- and *KRAS*-mutant lung adenocarcinomas, suggesting that tumors without mutational activation of these oncogenic pathways are more driven by copy number aberrations than of point mutations. The *TP53*-mutated tumors had the opposite pattern with more focal complex events in the tumors harboring a *TP53* mutation. This was consistent with the findings in the pan-cancer study by Ciriello et al., which found *TP53* mutations enriched in the C-class (copy number driven) tumors [7].

The aberrational pattern is similar across different studies of NSCLC tumors. Previous studies have shown that the expression of genes located in chromosomal regions involved in gains or losses varies consistently with the DNA copy number [48, 49]. We approached this by first investigating how the gene expression is affected by copy number alterations and secondly to study whether any known cellular pathways are overrepresented in the list of affected genes and hence probably regulated by copy number. Two of the most significantly affected pathways were the *mTOR*- and *EIF2*- signaling pathways, both related to the *PIK3CA* gene. Mutations of the *PIK3CA* gene occur in lung adenocarcinoma tissue, but is a relatively seldom event [50]. We found *PIK3CA* frequently gained and the gene expression significantly correlated with gain in copy number. Among the genes associated with the *mTOR*-pathway, forty-five were cis-genes. The expression of several important oncogenes such as *AKT1*, *AKT2*, and *KRAS* were positively correlated with the copy number in our analysis. The *mTOR* gene and several of its effectors (*RPS6KB1 (p70S6K)*, *RPS6,* and *EIF4G1*) were also altered. The PI(3)K-mTOR pathway was one of the key pathways found activated at a protein level in a large lung adenocarcinoma study by TCGA [51]. In this paper the activation of the pathway was partly explained by mutations (in *PIK3CA* or *STK11*), but some samples with increased pathway-activation lacked known underlying mechanisms. We suggest that altered expression of cis-genes affected by underlying copy number aberrations may increase the activity of this pathwayDrugs that target the mTOR pathway have shown interesting results in other cancer types [52], which highlight its clinical importance. Clinical trials in lung cancer targeting the PI(3)K-mTOR pathway have shown variable responses when given as monotherapy [53–55]. The lack of responses in some patients may be due to the complex regulation of the pathway and interplay with other oncogenic pathways [54].

The chromosomal structure in lung carcinomas is highly aberrant and copy number alterations in tumor or in cell-free DNA might predict response to immunotherapy in cancer patients [56]. The findings in this study encourage further research of whole genome copy number alterations and to increase the biological understanding and of therapeutic approaches targeting the PI(3)K-mTOR pathway.

## Conclusion

Knowledge of molecular alterations in cancer is rapidly increasing, and it can be challenging to get an overview of the information and interpret the biological relevance of the data. In this study we studied common copy number events including the genomic architecture in NSCLC tumors and differences between subgroups of samples. The *TP53*-mutated lung adenocarcinomas showed highly aberrant copy number profiles in both genome-wide and chromosome-arm analyses and TP53 mutation status should always be considered included in studies of copy number aberrations in tumors tissue. Furthermore, nearly all *TP53*-mutated tumors had lost the second *TP53* allele, and this was also a frequent event in the *TP53* wild type tumors. The arm-wide analysis with samples stratified by the *EGFR* mutation status, revealed differences particularly at chromosome arm 9q and 7p involving the *EGFR* gene. To better understand the potentially functional effects of copy number aberrations we performed integrative analyses including copy number and mRNA expression data. By this approach we identified cis-genes whose expression correlated with copy number and further were associated with important oncogenic pathways such as the PI(3)K-mTOR pathway.

## Supplementary Information

**Additional file 1.**

**Additional file 2.** Recurrent aberrant regions and associated genes.

## Data Availability

Gene expression data is available in GEO for public release, accession code: GSE66863. Other data will be made available from the corresponding author upon reasonable request.
