# Supplementary material for: Whole genome copy number analyses reveal a highly aberrant genome in TP53 mutant lung adenocarcinoma tumors
Source: BMC Cancer. 2021 Oct 9;21:1089. doi: 10.1186/s12885-021-08811-7 (PMC8501630; doi:10.1186/s12885-021-08811-7)
Supplement: Supplementary file 1 — Additional file 1. [file 12885_2021_8811_MOESM1_ESM.docx]

**Supplementary tables and figures:**

**Supplementary tables:**

Supplementary table 1:

| **Patient and tumor characteristics:** | |  |  |
| --- | --- | --- | --- |
|  |  | **Histology** |  |
| **Variable:** | **Adenocarcinoma** | **Squamous cell carcinoma** | **Large cell carcinoma** |
|  | n=154 | n=32 | n=4 |
| **Age at surgery (years):** |  |  |  |
| Mean: | 65.3 | 66.5 | 59.5 |
| Median: | 65.5 | 66 | 59.5 |
| Range: | 39-84 | 43-80 | 49-70 |
| **Sex:** |  |  |  |
| Females | 87 | 10 | 1 |
| Males: | 67 | 22 | 3 |
| **Smoking history:** |  |  |  |
| Current: | 52 | 10 | 2 |
| Former: | 82 | 21 | 2 |
| Never: | 20 | 1 | 0 |
| ***EGFR* mutation status:** |  |  |  |
| *EGFR* mutated: | 20 | 0 | 0 |
| *EGFR* wild type: | 132 | 32 | 4 |
| *EGFR* not tested: | 2 | 0 | 0 |
| ***KRAS* mutation status:** |  |  |  |
| *KRAS* mutated: | 53 | 2 | 0 |
| *KRAS* wild type: | 96 | 30 | 4 |
| *KRAS* not tested: | 5 | 0 | 0 |
| ***TP53* mutation status:** |  |  |  |
| *TP53* mutated: | 58 | 19 | 2 |
| *TP53* wild type: | 96 | 12 | 2 |
| *TP53* not tested: | 0 | 1 | 0 |
| **Stage:** |  |  |  |
| Ia: | 41 | 11 | 1 |
| Ib: | 46 | 10 | 1 |
| IIa: | 27 | 5 | 2 |
| IIb: | 11 | 2 |  |
| IIIa: | 26 | 4 |  |
| IIIb | 1 |  |  |
| IV: | 2 |  |  |
| **Adjuvant chemotherapy:** |  |  |  |
| Yes: | 45 | 10 | 2 |
| No: | 102 | 21 | 2 |
| Not known: | 7 | 1 |  |

Supplementary table 2:

Recurrent aberrant regions and associated genes.

Supplementary table 2 is a large table and is includes in a separate document.

Supplementary table 3:

| Type | Chromosome arm: | Number of genes: |
| --- | --- | --- |
| Gains: | 1q | 1041 |
|  | 3q | 191 |
|  | 5p | 184 |
|  | 6p | 595 |
|  | 7p | 471 |
|  | 7q | 360 |
|  | 8q | 485 |
|  | 14q | 42 |
|  | 17q | 667 |
|  | 19q | 32 |
|  | 20p/q | 420 |
| Losses: | 1p | 49 |
|  | 3p | 551 |
|  | 4p | 191 |
|  | 4q | 544 |
|  | 5q | 798 |
|  | 6q | 494 |
|  | 8p | 671 |
|  | 9p/q | 880 |
|  | 10q | 281 |
|  | 11p | 67 |
|  | 13q | 402 |
|  | 14q | 14 |
|  | 15q | 681 |
|  | 16q | 51 |
|  | 17p | 366 |
|  | 18q | 210 |
|  | 19p | 617 |
|  | 21q | 277 |
|  | 22q | 357 |
|  | X | 820 |
|  | Y | 71 |

Overview of chromosomal aberrations that occur in more than 30% of samples and number of genes located in the affected segments. More details are found in supplementary table 2.

Supplementary table 4:

| **Variable:** | **Genome-wide scores:** | **Arm-wise scores:** |
| --- | --- | --- |
| **Adenocarcinoma vs squamous cell carcinoma:** | None | 1q: higher scores on dev, gain and asym in AD |
|  |  | 3q: higher scores on var, dev, gain and asym in SCC |
|  |  | 5q: higher gain in AD |
|  |  | 6q: higher loss and LOH in AD |
|  |  | 12p: higher loss and LOH in AD |
|  |  | 19q: higher curv and LOH in AD |
| **Never-smokers vs. Current/former smokers:** | None | 2q: higher curv in former/current smokers |
|  |  | 3q: higher steep in former/current smokers |
|  |  | 5q: higher loss in former/current smokers |
|  |  | 7p: higher loss in former/current smokers |
|  |  | 12p: higher gain in former/current smokers |
| ***EGFR* mutation vs *EGFR* wt:** | None | 3p: higher curv in wild type |
|  |  | 4p: higher LOH in wild type |
|  |  | 5p: higher curv in wild type |
|  |  | 7p: higher loss in wild type |
|  |  | 9q: higher gain in wild type |
|  |  | 11q: higher var, steep, curv and LOH in wild type |
|  |  | 12p: higher curv in wild type |
| ***KRAS* mutation vs *KRAS* wt:** | *KRAS* wild type had higher score on steep and curv | 2p: higher steep and asym in wild type |
|  |  | 11p: higher var, steep, curv and gain in wild type |
|  |  | 11q: higher steep, curv, gain in wild type |
|  |  | 16p: higher loss in wild type |
|  |  | 16q: higher asym in wild type |
|  |  | 18q: higher steep and asym in wild type |
| ***TP53* mutation vs *TP53* wt:** | The *TP53* mutant had higher scores on all 8 variables | 1q: higher dev, gain in *TP53*-mutant |
|  |  | 3p: higher LOH in *TP53*-mutant |
|  |  | 4q: higher dev, LOH in *TP53*-mutant |
|  |  | 5p: higher dev, gain, asym in *TP53*-mutant |
|  |  | 5q: higher dev and LOH in *TP53*-mutant |
|  |  | 6p: higher dev, gain and asym in *TP53*-mutant |
|  |  | 7p: higher dev and gain in *TP53*-mutant |
|  |  | 7q: higher var, dev and gain in *TP53*-mutant var, dev and gain |
|  |  | 8p: higher var, dev, LOH and loss in *TP53*-mutant |
|  |  | 8q: higher dev and gain in *TP53*-mutant |
|  |  | 9p: higher dev in *TP53*-mutant |
|  |  | 10q: higher dev in *TP53*-mutant |
|  |  | 11p: higher var and dev in *TP53*-mutant |
|  |  | 11q: higher gain in *TP53*-mutant |
|  |  | 12q: higher LOH in *TP53*-mutant |
|  |  | 13q: higher dev, loss and LOH in *TP53*-mutant |
|  |  | 14q: higher var, dev and gain in *TP53*-mutant |
|  |  | 15q: higher dev and LOH in *TP53*-mutant |
|  |  | 16p: higher dev and var in *TP53*-mutant |
|  |  | 17p: higher var, steep, dev, loss and LOH in *TP53*-mutant |
|  |  | 17q: higher dev, gain and LOH in *TP53*-mutant |
|  |  | 18p: higher LOH in *TP53*-mutant |
|  |  | 18q: higher var, dev, loss and LOH in *TP53*-mutant |
|  |  | 19p: higher LOH in *TP53*-mutant |
|  |  | 19q: higher dev and LOH in *TP53*-mutant |
|  |  | 20q: higher dev, gain and asym in *TP53*-mutant |
|  |  | 22q: higher LOH in *TP53*-mutant |

This table shows an overview of differences between subgroups of samples, based on the comparison-analyses of genome-wide and arm-wise scores. *Var*: variation relative to median copy number, *steep*: steep transitions, *curv*: curvature or oscillation, *dev*: deviation from ploidy, *gain*: gain relative to ploidy, *loss*: loss relative to ploidy, *loh*: LOH related allelic skewness, and *asym*: non-LOH related allelic skewness

Supplementary table 5:

| **Top canonical pathways** | **Genes in genelist/genes in pathway** | **p-value** | **BH corrected p-value** |
| --- | --- | --- | --- |
| Protein Ubiquitination Pathway | 73/255 | 1.47·10^-11^ | 7.08·10^-9^ |
| EIF2 signaling | 57/181 | 4.23·10^-11^ | 1.02·10^-8^ |
| Estrogen Reseptor signaling | 41/127 | 9.82·10^-9^ | 1.58·10^-6^ |
| Regulation of eIF4 and p70S6k signaling | 44/145 | 2.36·10^-8^ | 2.85·10^-6^ |
| tRNA charging | 19/39 | 5.78·10^-8^ | 5.58·10^-6^ |
| Glucocorticoid Receptor Signaling | 63/275 | 2.73·10^-6^ | 2.2 ·10^-4^ |
| 3-phosphoinositide biosynthesis | 40/153 | 6.73·10^-6^ | 4.64·10^-4^ |
| Mitochondrial dysfunction | 42/169 | 1.55·10^-5^ | 8.82·10^-4^ |
| mTOR signaling | 45/186 | 1.64·10^-5^ | 8.82·10^-4^ |
| NGF signaling | 30/107 | 2.17·10^-5^ | 1.05·10^-3^ |
| **Top molecular and cellular functions** | **Molecules** | **p-value** |  |
| RNA post-transcriptional modification | 129 | 1.65·10^-2^ to 9.73·10^-28^ |  |
| Post-translational modification | 355 | 1.04·10^-2^ to1.44·10^-17^ |  |
| Gene expression | 509 | 1.30·10^-2^ to 1.65·10^-15^ |  |
| Molecular transport | 144 | 1.27·10^-2^ to 1.08·10^-14^ |  |
| Protein trafficking | 143 | 1.27·10^-2^ to 1.08·10^-14^ |  |
| **Top associated diseases** | **Molecules** | **p-value** |  |
| Infectious diseases | 385 | 1.65·10^-2^ to 1.75·10^-18^ |  |
| Organismal Injury and abnormalities | 2203 | 1.65·10^-2^ to 1.59·10^-11^ |  |
| Cancer | 2185 | 1.65·10^-2^ to 1.73·10^-10^ |  |
| Metabolic disease | 70 | 1.65·10^-2^ to 3.52·10^-8^ |  |
| Gastrointestinal disease | 1496 | 1.65·10^-2^ to 6.51·10^-8^ |  |

Results from the core analysis in Ingenuity Pathway Analysis with the 22,076 cis-genes as input genes. This table shows the top canonical pathways and the fraction of cis-genes compared to total genes in this pathway, the top molecular and cellular functions and the top diseases associated with the cis-genes.

p-value: raw p-value calculated with Fisher’s exact test

BH corrected p-value: p-value after correction for multiple comparisons with the Benjamini-Hochberg method.

**Supplementary figures:**

Supplementary figure 1:


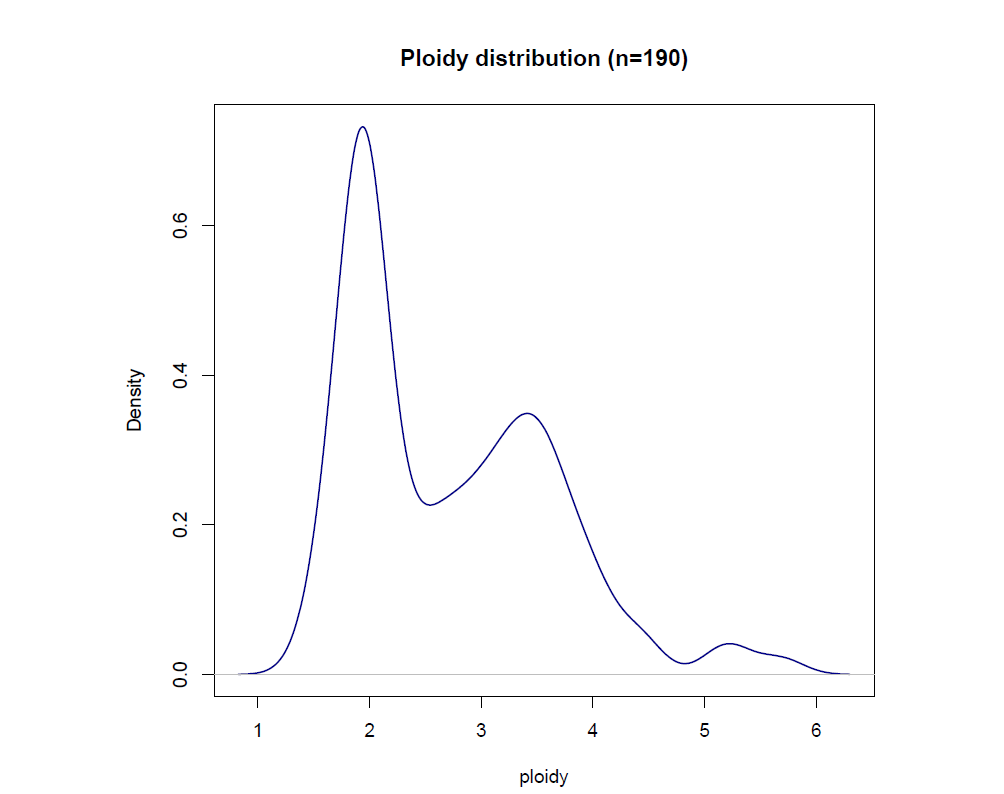


This plot shows the distribution of ploidy of all samples included in the analysis. The x axis shows the estimated ploidy of the samples with a ploidy of 2 equals a diploid genome, 3 a triploid genome and 4 a tetraploid genome. The y-axis shows the fraction of samples. In this plot we see a peak of samples with a ploidy close to 2N, a second peak at 3.5 and a third small peak at 5.5.

Supplementary figure 2:

2a)

2b)

Two examples of copy number profiles, and arm-wise heatmaps for samples included in the analyses. The CN curve shows the total copy number, with the dashed horizontal line indicating the ploidy. The allele fraction curve shows the distribution and the fraction of the alleles, and 0.5 reflects equal allelic copy numbers. Values above and below 0.5 reflect the fraction of the number of copies of the most frequent and the least frequent allele, relative to total copy number. The heatmap show the scores from the arm-wise analysis, with one score for each index on every chromosome arm. The color intensities indicate the value of the scores; the stronger the color, the larger the score. The first example (a) has generally high scores at all eight indices, and this was captured both in the arm-wise and the genome-wide analyses. This example was from the tumor of a 67-year-old male smoker with a stage Ia lung adenocarcinoma tumor harbouring a TP53-mutation. The second example (b) has a less complex profile, captured both in the arm-wise and genome-wide analyses. This was from a 69-year-old female former-smoking patient with stage Ia lung adenocarcinoma disease without mutations of the EGFR, KRAS or TP53 genes*.*

Supplementary figure 3:


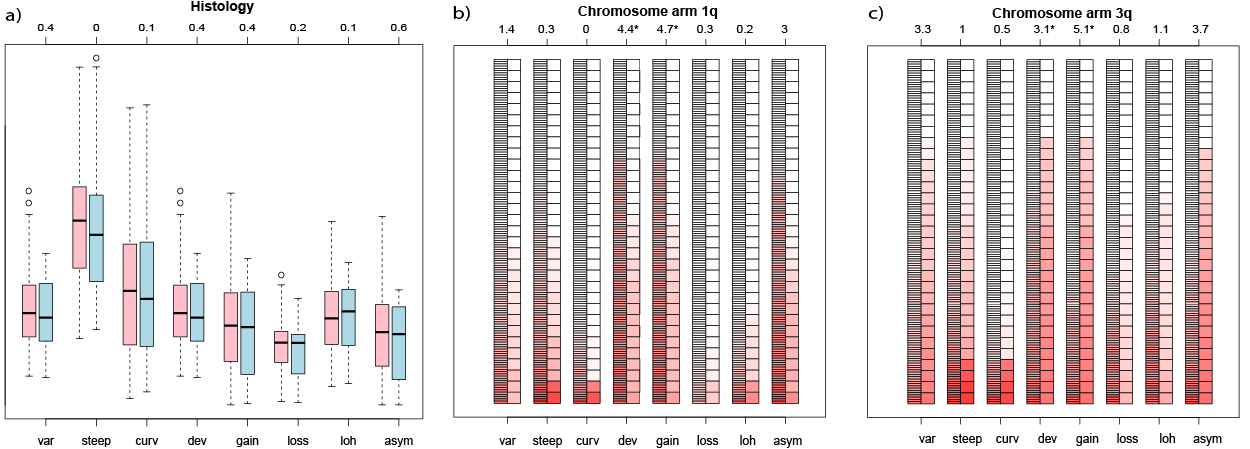


a)

Box-plot showing the genome-wide scores of all eight indices for adenocarcinomas (pink boxes) and for squamous cell carcinomas (blue boxes). The p-values (-log10) from the comparison analyses are shown above each column and a value > 2 was considered as significant after multiple testing corrections in the genome-wide analysis. None of the scores were significantly different in this analysis.

b and c)

The arm-wise scores at chromosome arm 1q (b) and 3q (c) from the arm-wise comparison analysis between lung adenocarcinomas (left in each column) and squamous cell carcinomas (right in each column). The scores for each sample are colored by intensity. The indices are named below each column and the p-values (-log10) are shown above each column with a value > 3 as significant after multiple testing corrections in the arm-wise analysis.

Supplementary figure 4:
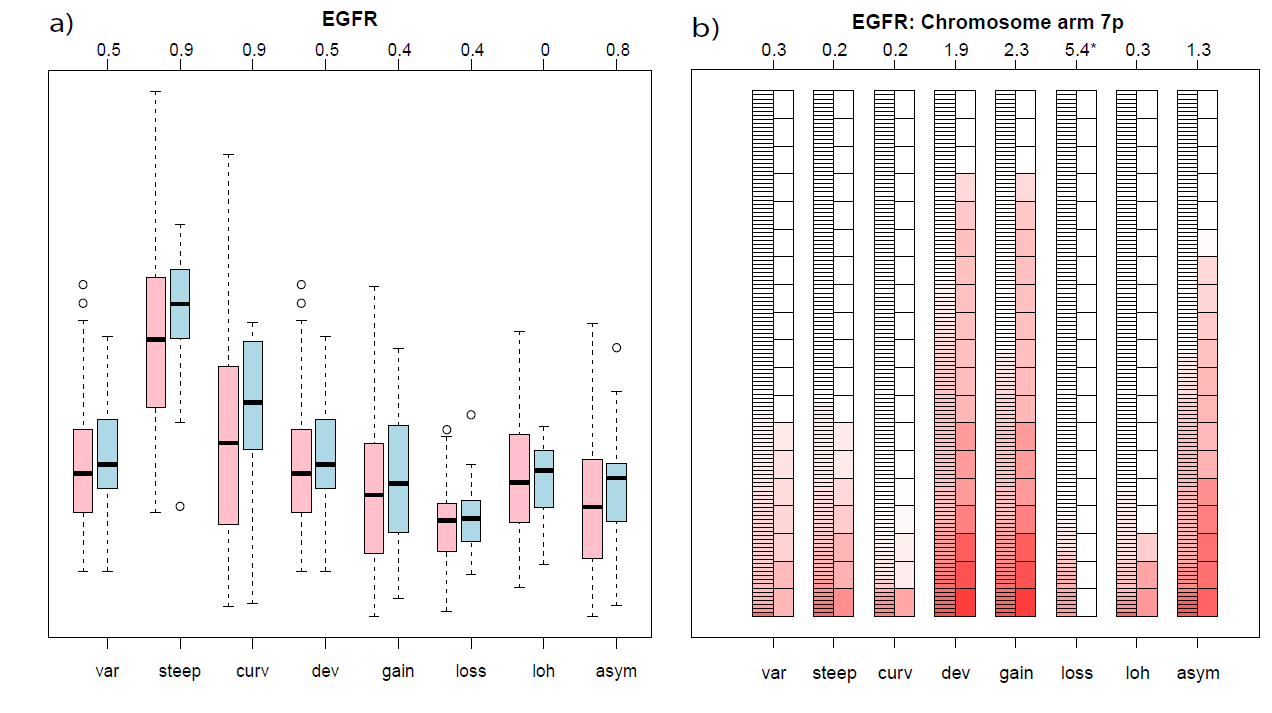


a)

Box-plot showing the genome-wide scores of all eight indices when we compare the EGFR wild type tumors (pink boxes) with EGFR-mutated tumors (blue boxes). The p-values (-log10) from the comparison analyses are shown above each column and a value > 2 was considered as significant after multiple testing corrections. None of the scores were significantly different in this analysis.

b)

The arm-wise scores at chromosome arm 7p showing the eight indices for the EGFR wild type tumors at the left and the EGFR mutated tumors at the right of each column. The scores for each sample are colored by intensity. The p-values (-log10) from the comparison analyses are shown above each column and a value > 3 was considered as significant after multiple testing corrections.

Supplementary figure 5:


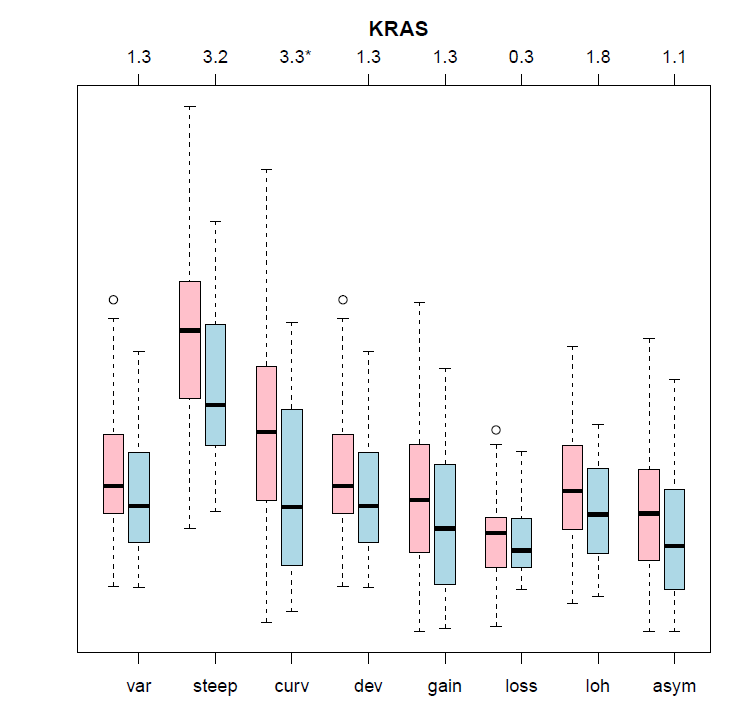


This figure shows the distribution of the genome-wide scores between the KRAS wild type tumors (pink boxes) and KRAS-mutated tumors (blue boxes). The p-values (-log10) from the comparison analyses are shown above each column and a value > 2 was considered as significant after multiple testing corrections in the genome-wide analysis.

Supplementary figure 6:

**
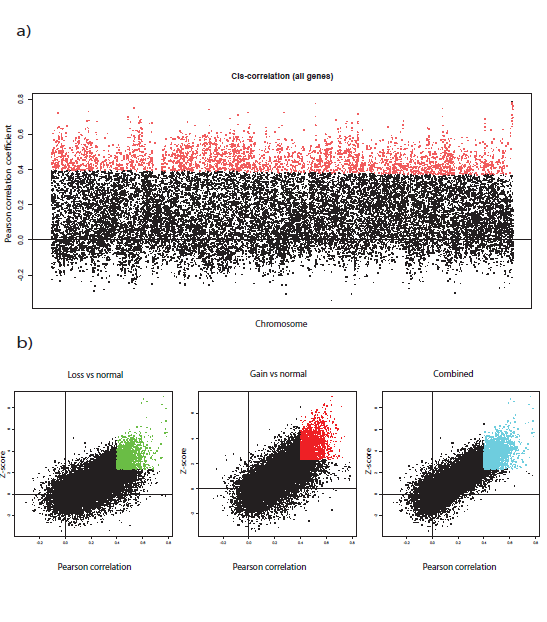
**

a) This plot shows the correlations between copy number and mRNA expression for all 22,076 genes. The Pearson correlation coefficients are plotted as a function of the genomic position for all genes included. The red dots are genes with Pearson correlation coefficient > 0.4.

b) Each panel displays Z-score versus Pearson cis-correlation for all 22,076 genes. In left and middle panel, the Z-score is based on the p-value from the t-test comparing expression in tumors with loss with tumors with no aberration and gain with tumors with no aberration. The genes marked in green have high cis-correlation (Pearson’s r >0.4) and differential gene expression in samples with loss vs samples with normal copy number. The genes marked in red in the middle panel have both high cis-correlation and differential expression in samples with gain vs normal copy number. The right panel uses, for each gene, the larger of the two preceding Z-scores. Each Z-score is found as Z = -F^-1^ (Pval), where F is the standard normal distribution function.
